# Building trusting relationships in teams to support evidence use and implementation in human services: feasibility and acceptability of a training and coaching approach

**DOI:** 10.3389/frhs.2024.1353741

**Published:** 2024-12-10

**Authors:** Allison Metz, Todd M. Jensen, Lacy Dicharry, Amanda B. Farley

**Affiliations:** ^1^ School of Social Work, University of North Carolina at Chapel Hill, Chapel Hill, NC, United States; ^2^ School of Education, University of North Carolina at Chapel Hill, Chapel Hill, NC, United States; ^3^ College of Human Sciences and Education, Louisiana State University, Baton Rouge, LA, United States

**Keywords:** evidence use, implementation practice, implementation science, implementation support, trusting relationships

## Abstract

**Background:**

Professionals who provide implementation support in human service systems describe relationships as being critical to support evidence use; however, developing trusting relationships are not strongly featured in implementation science literature. The aims of this study were to (a) assess the feasibility and acceptability of a theory-driven training and coaching approach for building trusting relationships among members of an implementation team who were supporting the implementation of an evidence-informed program in a public child welfare system in the United States and (b) gauge the initial efficacy of the approach in terms of the development of trusting relationships and subsequent implementation outcomes.

**Methods:**

Consistent with a convergent mixed-methods approach, we collected both quantitative and qualitative data to address our research questions. Quantitative methods included an adapted version of the Trusting Relationship Questionnaire, a seven-item measure of psychological safety, and items designed to measure acceptability of the training. Qualitative data were collected through semi-structured interviews with participants.

**Results:**

Sixteen individuals participated in the program, consisting of a kick-off training event, five monthly training modules, and five monthly coaching sessions with implementation team leads. Session attendance rates and self-reported satisfaction highlight the general feasibility and acceptability of the training and coaching approach. On average, participants also reported significant increases over time in their perceptions that they were trusted by their team. Results from in-depth interviews further indicated the efficacy of the program in terms of cultivating trust among team members and promoting several elements that were theorized to link trusting relationships to implementation outcomes.

**Discussion:**

Findings suggest the training and coaching approach for trust building was acceptable and feasible. Additionally, results indicate the value of the approach in building trust among implementation partners to increase commitment to implementation efforts, promote a culture of learning and psychological safety, and increase participants' sense of capability and motivation for supporting implementation.

## Introduction

Implementation science seeks to integrate research and practice in ways that improve outcomes for people and communities (1). The research component of implementation science identifies and evaluates approaches used to translate research evidence into practice settings. Implementation research has yielded a multitude of concepts, frameworks, models, and strategies for supporting implementation efforts in real world contexts. Change efforts are also strengthened by implementation practice, which involves applying and adapting these approaches to ensure that implementation efforts are sensitive to context and relevant for the people involved (2).

There is an increasing call for the advancement of a workforce capable of integrating implementation research—concepts, models, frameworks, and strategies—into practice to support evidence use, advance equity, and achieve improved population outcomes (1, 3–5). The importance of workforce development for implementation practice has been noted as a “grand challenge” in human services in recognition of this need (6, 7), as training programs have lagged behind the demand for an implementation workforce. Furthermore, the shortage of individuals trained in the practice of knowledge translation and implementation has been cited as a reason for failure to optimize the use of evidence to advance equity and improve population outcomes (8).

Implementation support practitioners (ISP) represent one approach for building implementation capacity in human service organizations and systems. ISPs are professionals who help systems and service providers implement research-supported practices, policies, and programs, and sustain and scale research evidence for population impact (9). They can reside *outside* the service systems they work in but may also operate from *within* a service system when those systems have internal work units specifically designed to support innovation, implementation, improvement and/or scaling efforts. ISPs may lead implementation teams, or provide external support to teams, and they can support both public and private service agencies. Although such internal leaders and staff may not refer to themselves as ISPs or implementation specialists, they often do the work of supporting implementation. In the context of the current study, we were interested in building the skills of these leaders and staff in public systems. Recent literature has proposed to consolidate the terms used for professionals who support implementation—facilitators, coaches, consultants, technical assistance providers—under a single term of implementation support practitioners (9). Specifically, we partnered with an implementation team in a public child welfare system in a northeastern state of the United States (U.S.), which provides an array of services to ensure the state's children, youth, and families are safe, healthy, and connected. Use of evidence is a core value in this context, reflected through investment in evidence-based and evidence-informed practices as well as the use of evidence to inform decision-making by implementation teams charged with overseeing implementation of those practices.

A range of competencies are emerging to support the uptake of research evidence and to guide the development and day-to-day work of professionals who provide implementation support in human services sectors. Metz and colleagues have identified and operationalized a set of guiding principles (i.e., foundational *attitudes* for approaching work, decision-making, and interactions) and competencies (i.e., necessary *abilities* to effectively support the sustained uptake of research), and recently validated them through an international study of ISPs (10–12). Defining the principles and competencies of professionals who support implementation also provides an opportunity to acknowledge specific skills central to implementation support. The current study focuses specifically on the implementation practice skill of *developing trusting relationships*.

### The role of trusting relationships in supporting evidence use and implementation

**Figure 1:**
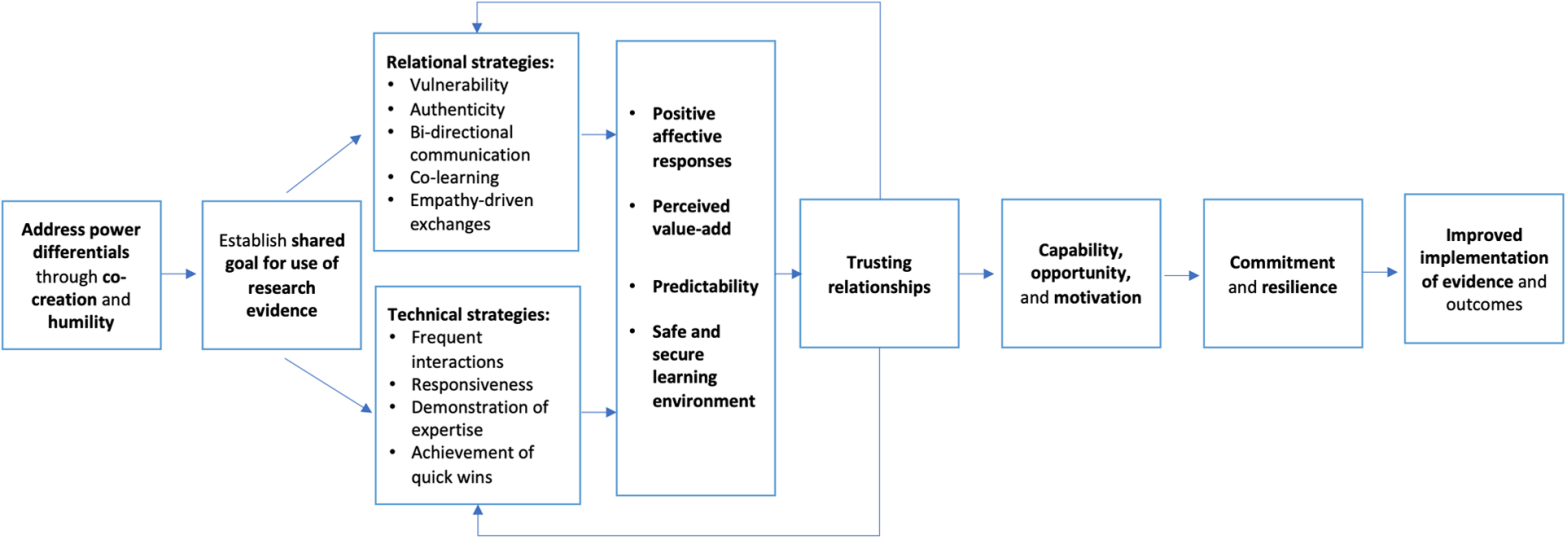
Theoretical model of how to build trusting relationships to support implementation and evidence use. Figure adapted with permission from Metz, Jensen, Farley, Boas, Bartley & Villodas (2022).

Although relationship-building is a commonly acknowledged task of professionals supporting implementation and evidence use (13–18), few studies have explored it in depth (19–21), limiting our theoretical and practical understanding of how relationships among ISP teams and implementation partners can be effectively built and why they are important. Drawing from existing literature and theory (e.g., relational cohesion theory, cultural exchange theory, relational cultural theory), Metz and colleagues (2022) recently proposed an integrated theoretical model that features (a) plausible strategies for trust-building and (b) specific mechanisms linking trusting relationships and improved implementation or evidence use (see Figure 1).

Relational cohesion theory offers an important theoretical basis for connecting relationships to the successful and sustainable use of evidence to improve outcomes. Relational cohesion is defined as the perception by individuals in an exchange relation that their relationship is a unifying element or force in the social situation (22, 23). Such perceptions lead to higher levels of commitment and collectivistic behavior. Relational cohesion theory posits that the relationships that emerge from positive affective experiences are valuable in and of themselves and contribute to relational cohesion and increased resilience and commitment in the face of challenges. Relational cohesion theory is aligned with cultural exchange theory (24) and with literature from implementation science on the role of research-practice partnerships in social work. Palinkas and colleagues describe cultural elements of successful partnerships including flexibility and sensitivity to the needs of individuals in the partnership, openness and honesty associated with building and maintaining trust, and humility and tolerance in service to mutualism and shared understanding of the work (25).

Relational cohesion theory also seeks to explain the conditions under which positive emotions are experienced within the exchange relation. In the case of evidence use in child welfare, relational cohesion theory can be used to explain the conditions under which instrumental exchanges of implementation support become more affective, emotional, and meaningful to partners at the implementing site, and what type of activities lead to relational cohesion within implementation teams and among community partners.

Relational cultural theory (26) presents a fuller picture of the types of strategies needed to promote positive affect and increased resilience and commitment and highlights the role of empathy in supporting the growth-promoting relationships needed for implementation. Metz and colleagues identified empathy, curiosity, and commitment as active ingredients that support evidence use (12). Bürhmann and colleagues identified seven attitudes for supporting evidence use: professional, motivating, empathetic, collaborative, authentic, flexible and creative, and honest (27).

The integrated theoretical model developed by Metz and colleagues foregrounds (a) underlying assumptions about the competencies needed to build trusting relationships, (b) the role trusting relationships play in behavior change (9, 28), and (c) how changes in individual and team behavior can contribute to use of evidence in public systems (18). Figure 1 illustrates underlying assumptions related to the theoretical model that guided the current study and associated research questions. Key underlying assumptions are as follows: (a) implementation partners must first establish a shared goal for use of research evidence; (b) team members can feasibly be trained and coached to use relational strategies (e.g., empathy-driven exchanges, bi-directional communication) and technical strategies (e.g., responsiveness, frequent interactions); (c) the use of specified relational and technical strategies, which comprise the overall trust-building strategy, will produce among team members positive affective responses, perceived value-add in the partnership, predictability, and a psychologically safe and secure learning environment (29–31), leading to trusting relationships and relational cohesion; and (d) trusting relationships among team members will lead to increased capability, opportunity, and motivation to use evidence for decision-making, and an increased commitment to implementation efforts and ability to successfully withstand adversity over time (i.e., resilience), leading to sustainable implementation of evidence use in the public agency.

We also acknowledge that implementation activities are influenced by and contribute to power structures. Stanton and colleagues describe three types of power including discursive power that is enacted by how problems are defined, epistemic power that influences whose voices are valued in decision-making, and material power that is created by resource allocation (32). The theoretical underpinnings of the current model highlight the role of interpersonal trust in contributing to implementation outcomes and is limited in its ability to fully address power dynamics in implementation efforts. However, trust-building activities that promote psychologically safe environments can recalibrate epistemic power in the implementation setting through more inclusive decision-making processes on teams.

### Strategies to build trusting relationships among implementation team members

Implementation science has focused on the role of implementation support teams in facilitating the achievement of implementation and population outcomes. Metz and Bartley define an implementation team as a “group of stakeholders that oversees, attends to, and is accountable for facilitating key activities in the selection, implementation, and continuous improvement of an intervention” (33). The role of trusting relationships is foundational for implementation teams that collectively leverage members’ diverse skills and perspectives and ensure the inclusion of all team members in activities such as communication, problem solving, and data-driven decision-making.

Building effective implementation teams requires new thinking on how to facilitate effective team meetings, support co-learning among team members, and develop a sense of mutual accountability for progressing implementation in complicated systems (33). Implementation teams are responsible for both taskwork and teamwork—meaning, team members need to be effective at working together in order to successfully complete tasks.

There is recent interest in strategies that target team effectiveness and contribute to how well teams work together (34). Specifically, successful implementation teams have a common goal and are cumulatively responsible for ensuring completion of necessary tasks that involve high interdependence (33). This high level of interdependence requires relationships, trust, and psychological safety (35). Research evidence shows strategies that enable successful teams include promoting shared mental models, bi-directional communication, trust, and shared leadership (36). Because of this, implementation teams are an excellent structure for testing the use of relational and technical strategies to build trust and foster environments that promote psychological safety (37).

### Current study

Professionals who provide implementation support describe relationships as being at the heart of what they do to support evidence use (17). However, developing trusting relationships and addressing power differentials are not strongly featured in implementation science literature (18). A recent study highlighted *trusting relationships* as necessary to enable successful implementation and sustained evidence use (17). Study participants, which included professionals with extensive experience supporting the use of evidence-based practices in child and family services, emphasized with a striking amount of uniformity that high-quality relationships between those providing implementation support and partners in child and family services was a—if not *the*—critical factor for achieving implementation results.

The current study assessed the feasibility of developing and delivering a training and coaching curriculum for implementation partners within a public child welfare system for building trusting relationships. A public child welfare setting is a particularly interesting setting for this current study, where implementation partners may historically not collaborate or trust each other. For example, private service providers may see themselves as competitors for service delivery contracts from the public system (38). Further, the public agency serves in the role of funder and monitor, setting up a power dynamic between private service providers and the public agency. In the current study, statewide implementation required collaboration and relationship-building among participants from the public system and private service providers in order to effectively scale and sustain an evidence-based model.

The study assessed whether building trusting relationships contributes to short-term outcomes such as relational cohesion on implementation teams; capability, opportunity, and motivation to use evidence; and commitment and resilience for implementation. Trusting relationships are a strategy used to improve the use of research evidence and are defined as relationships centered in vulnerability where the beliefs or expectations of individuals in the relationship are that actions will cause no harm and will provide benefit (39). With this definition, trusting relationships are conceptualized as a strategy for improving implementation. However, trusting relationships can also be conceptualized as a moderator of associations between commonly applied implementation strategies and implementation outcomes (18). We conceptualize evidence use as the instrumental use of research evidence by implementation teams to make decisions and improve child welfare practice. The current study addressed the following four specific research questions:

(1) What is the feasibility of developing and delivering a pilot training and coaching curriculum that builds the skills of leadership and staff in a public child welfare system who support implementation of evidence-informed initiatives to foster trusting relationships among each other and with community partners? (RQ1)
(2) What is the acceptability of the pilot training and coaching curriculum among leadership and staff in a public child welfare system who serve on an implementation team and support implementation of evidence-informed initiatives to foster trusting relationships among each other and with community partners? (RQ2)
(3) To what extent does the pilot training and coaching program demonstrate contributions to the development of trusting relationships among leadership and staff and with community partners who serve on implementation teams to support implementation of evidence-informed initiatives? (RQ3)
(4) In what ways does the development of trusting relationships among leadership and staff and with community partners who serve on an implementation team demonstrate contributions to the emergent and intended use of evidence for decision-making and implementation of evidence-informed initiatives? (RQ4)

## Methods

### Development and delivery of training/coaching approach

#### Project partners and setting

To support the current study, we partnered with a public child welfare agency in a northeastern state in the U.S. Specifically, we engaged with an implementation team charged with developing a statewide implementation strategy and guidance for local-level implementation of a youth-centered, strengths-based initiative that leverages peer navigators to help increase child-welfare-involved youth's ability to articulate and work toward their goals, interact with professionals, and initiate connections to resources. The implementation team was comprised of co-leads and staff from within the public child welfare agency, model developers, and staff at service provider agencies. Across the duration of the project, between 11 and 16 team members were present during each training module (described in more detail below). Although there was some slight fluctuation in team membership given the dynamic needs of the team and overall project (e.g., onboarding new staff in preparation for regional collaboration teams to engage in local implementation/evidence use efforts), team membership at baseline included six child welfare agency staff/co-leads, four model developers, and six staff from service delivery agencies.

#### Content and format

**Figure 2:**
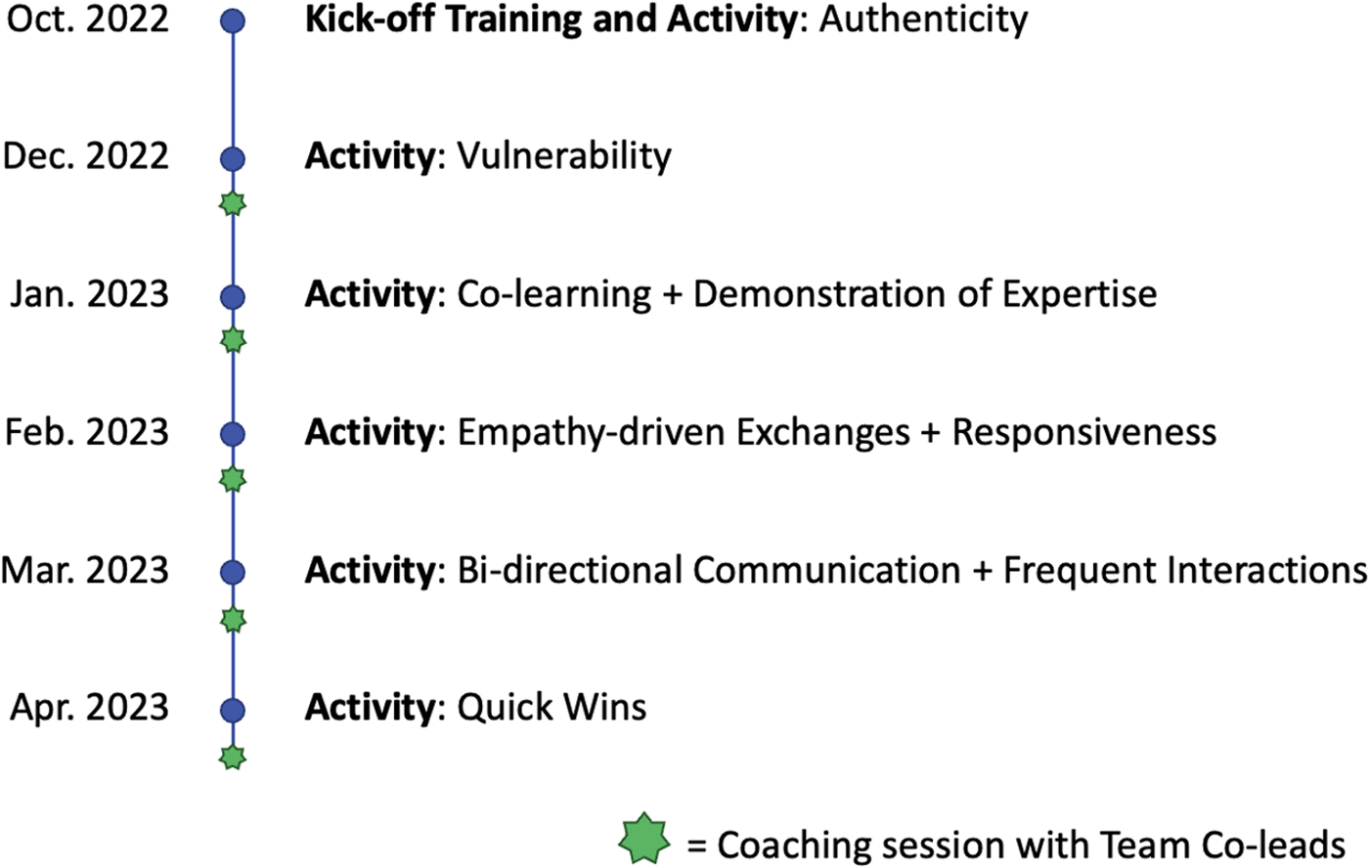
Visual overview and sequence of training and coaching components.

Content for the training and coaching approach was developed based on its alignment with recent theorizing focused on how trusting relationships can be built to support implementation and evidence use in human-service settings (18). As shown in Figure 1, both relational and technical strategies can support trust-building in teams. Relational strategies can be undertaken to build trust through strengthening the quality, mutuality, and reciprocity of interactions among team members. Relational strategies include (a) vulnerability (engaging in relational uncertainty, risk, and emotional exposure); (b) authenticity (approaching interactions openly, honestly, and in alignment with values); (c) bi-directional communication (establishing effective feedback loops); (d) co-learning (inviting and valuing the sharing of individual experiences to support learning); and (e) empathy-driven exchanges (seeking to understand the perspectives and emotional experiences of others). Technical strategies also can be undertaken to build trust by demonstrating the knowledge, reliability, and competency to support the goals of the team. Technical strategies include (a) frequent interactions (emphasizing meeting frequency over duration); (b) responsiveness (acknowledging and responding to requests as quickly as possible and tailoring support); (c) demonstration of expertise (sharing accurate and credible information); and (d) achievement of quick wins (celebrating early signs of progress and sharing widely). A series of interactive, synchronous, facilitated modules were developed, each with a focus on a particular strategy or set of strategies for trust-building. Figure 2 provides an overview of each module and the sequence by which they were delivered. Each model included a mixture of didactic content delivery and application activities, consistent with adult learning principles (40). Additional details about specific trust-building strategies and exercises used in training sessions are available in the Supplementary Materials associated with this article.

Starting in October 2022, we delivered a kick-off training and activity that was 1.5 h in duration. This module focused on authenticity as a relational strategy and provided participants with an overview of the full training and coaching approach. The remaining modules, delivered monthly between December 2022 and April 2023, were each 30–45 min in duration and embedded within existing team meetings. Our intention was to reduce participant burden as much as possible and remove barriers to participant engagement. The December 2022 module focused on vulnerability; the January 2023 module focused on co-learning and demonstration of expertise; the February 2023 module focused on empathy-driven exchanges and responsiveness; the March 2023 module focused on bi-directional communication and frequent interactions; and the April 2023 module focused on quick wins. All modules were delivered remotely via the Zoom web-conferencing platform.

In between the modules delivered from December 2022 and April 2023, we facilitated coaching sessions with three team co-leads, who were senior managers in the public system with oversight for the initiative. The coaching sessions were approximately 45 min and provided space for team leads to process new learnings and identify suitable opportunities to continue applying those learnings within the larger team over time. All coaching sessions were facilitated remotely via the Zoom web-conferencing platform.

### Evaluation

#### Data collection and measures

Consistent with a convergent mixed-methods approach, we collected both quantitative and qualitative data to address our research questions (41). A convergent mixed-methods approach was optimal because our research questions warranted the joint consideration of quantitative and qualitative data, whereby the quantitative data served a relatively descriptive function, and the qualitative data served a relatively interpretive function (42). In terms of quantitative data related to the training modules, we issued a web-based survey (*via* Qualtrics) at baseline (2–4 weeks prior to the kick-off training), immediately following the kick-off training, and following each monthly module (7 surveys total). The first survey provided informed consent materials for research purposes, as approved by our University Institutional Review Board (IRB #21-3172; determined to be exempt from further review according to the regulatory category cited above under 45 CFR 46.104), and enabled respondents to identify their role (i.e., child welfare agency staff, model developer, staff at service provider agency). Each survey also enabled respondents to insert a unique identifier (ID number), which was issued to them at random and managed by one member of the research team who did not have access to any collected data in Qualtrics. The unique identifier allowed us to connect responses over time from the same individual—a feature that facilitated our data analysis.

Each survey included an adapted version of the Trusting Relationship Questionnaire (43), which included two eight-item subscales—one which measured respondents’ perceptions of others on the team (e.g., “Do team members share information openly?” “Do team members initiate contact with you in times of need?” “Do team members consider your point of view?”; *α*=0.91) and another that measured respondents' perceptions of self in relation to members of the team and another (e.g., “Do you seek out advice from members of your team?” “Do you talk to members of your team about work-related problems?” “Do you share information openly with members of your team?”; *α* = 0.86). Response options ranged from 1 = Never to 5 = Very Frequently, and all items were coded such that higher values indicated higher levels of trust. Respondents could also indicate whether an item did not apply to their situation. In preparation for analyses, average scores were estimated for each subscale.

A seven-item measure of psychological safety (29) was also issued at baseline (*α* = 0.79) and following the final training module (*α* = 0.79). Psychological safety exists when people feel safe enough to take interpersonal risks, speak up, voice concerns, ask questions, and share ideas (44). Items included the following: “If you make a mistake on this team, it is often held against you,” “Members of this team are able to bring up problems and tough issues,” and “It is safe to take a risk on this team.” Response options ranged from 1 = Very Inaccurate to 6 = Very Accurate, and all items were coded such that higher values indicated higher levels of perceived psychological safety. In preparation for analyses, we estimated an average score for psychological safety at both time-points.

Surveys issued following the delivery of training modules also included items intended to measure acceptability of the training through participants' reactions to the content and learning experiences. Acceptability was defined as satisfaction with the training, and feasibility was assessed through attendance, delivery time, and perceived fit of the training (45). Specifically, the following two closed-ended items were provided following each training session, “I am satisfied with the training session just delivered,” and “I would recommend this training session to peers or colleagues.” Response options ranged from 1 = Strongly Disagree to 6 = Strongly Agree. One open-ended survey item was also provided, allowing respondents to provide context for their closed-ended responses and to share any other feedback. In terms of the delivery of training sessions, we monitored the delivery time for each training to assess the feasibility of integrating training into the activities of the implementation team. With respect to quantitative data linked to coaching sessions, we issued the following two closed-ended items following each coaching session for team co-leads, “I am satisfied with coaching session just delivered,” and “I would recommend this coaching session to peers or colleagues.” Response options ranged from 1 = Strongly Disagree to 6 = Strongly Agree. One open-ended survey item was also provided, allowing respondents to provide context for their closed-ended responses and to share any other feedback.

Turning to qualitative data, we conducted semi-structured, in-depth interviews with willing participants (*n* = 7) following the completion of all training and coaching sessions. Interviews were conducted by the first, second, and third authors of the study. Interviews took place via the Zoom web-conferencing platform and were audio recorded. Coding and theme development was conducted by the first, second, and fourth authors of the study (using Microsoft Word). Authors were not involved with assessing implementation progress or evaluating the evidence-based program, nor did the authors have any requirements to report on team progress to agency leadership. The interviews were approximately 60 min in duration and captured information about participants’ general experiences with the training and coaching approach and the potential impacts of the approach on implementation and evidence use. Sample questions included: “Consider how you felt about the training sessions. What emotion words best describe your experiences?” “Overall, how did the training session influence your team's ability to build relationships with each other?” “In what ways did relationships among team members help the team support implementation of the peer mentoring program?” “Can you share an example of a decision that the implementation team made and describe the process by which the decision was made?” A subset of questions related to coaching sessions were reserved only for team co-leads who participated in an interview. For example, “What impacts did the coaching sessions have on anything we discussed so far?” the full interview protocol is available in the Supplementary Materials associated with this article.

#### Data analysis

Descriptive analyses of closed-ended feedback items and module attendance were conducted to yield answers to RQ1 (feasibility) and RQ2 (acceptability). For module attendance, we estimated counts of all participants for each training module. For closed-ended feedback items linked to both training sessions and coaching sessions, we calculated the percentage of relevant respondents who indicated agreeing or strongly agreeing with the item statements. To address RQ3 (potential impact of approach on trusting relationships), we employed multilevel mixed-effects regression analysis with longitudinal observations (level 1) nested within individuals (level 2). Two distinct models were estimated—one focused on participants' perceptions that they were trusted by their team and another focused on participants' trust toward their team members. Both models controlled for participant role and baseline levels of perceived psychological safety (mean-centered). The models also focused on participants who were involved beginning at baseline data collection, resulting in an analytic sample of 15 individuals with 88 observations total (average of 5.9 data-points per participant out of 7 possible).

In terms of the analysis of in-depth interviews (audio recordings were transcribed verbatim in preparation for analysis), we employed principles of reflexive thematic analysis by engaging in the following key phases of analysis (a) familiarization (i.e., foundational and thorough engagement with the interview data), (b) organic coding process (i.e., open-coding process unburdened by strict consistency across coders, enabling the emergence of codes from various vantage points to inform theme development), (c) initial theme generation using codes, and (d) testing initial themes against the data and refining themes as needed (42). We prioritized the coding of content reflecting concepts represented in the theoretical model of trust-building (See Figure 1). As applicable, we also coded and developed themes representing novel insights that fell outside the theoretical model. On this front, we attended to any information related to how participants experienced the training and coaching approach.

## Results

Turning first to module attendance as an indicator of feasibility and acceptability (RQ1 and RQ2), attendance across all modules (including the initial kick-off event) ranged between 69% (Input 3) and 94% (Input 1). Overall, given the numerous other work demands and potential scheduling conflicts, these attendance rates provide some evidence for the feasibility and acceptability of the training and coaching approach. All trainings sessions were completed in the time allotted (40 min) by the public system during regularly scheduled implementation team meetings. The timeframes for training delivery demonstrate the feasibility of integrating specialized training on trust-building into business-as-usual activities for teams in public systems without disrupting their required activities related to the implementation, improvement, and scaling of evidence-informed programs and practices.

Table 1 displays the percentage of respondents who agreed or strongly agreed (hereafter referred to as “agree” or “agreement”) with feedback statements regarding each training session. Between 86% and 100% of respondents agreed that they were satisfied with particular training sessions, with an overall percent agreement of 88% across all training sessions. Between 80% and 100% of respondents agreed that they would recommend particular training sessions to peers or colleagues, with an overall percent agreement of 84% across all training sessions. The strongest levels of agreement were yielded for the kick-off training, which was focused on authenticity as a relational strategy for trust-building.

**Table 1:** Reactions to training sessions.

Session	*n*	I am satisfied with the training session just delivered	I would recommend this training session to peers or colleagues
		% Agree/Strongly agree	% Agree/Strongly agree
Kick-off training (authenticity)	14	100%	100%
Input 1: Vulnerability	14	86%	85%
Input 2: Co-learning & demonstration of expertise	12	92%	83%
Input 3: Empathy-driven exchanges and responsiveness	10	90%	80%
Input 4: Bi-directional communication and frequent interactions	14	100%	86%
Input 5: Quick wins	15	87%	80%
Completion		Overall, I am satisfied with the full training	I would recommend this full training to peers or colleagues
		% Agree/Strongly Agree	% Agree/Strongly Agree
Overall	14	88%	84%

*n* represents the number of valid responses provided by participants.

Table 2 displays the percentage of respondents who agreed with feedback statements regarding each coaching session. Except for the coaching session focused on vulnerability, 100% of respondents agreed that they were satisfied with each coaching session and that they would recommend the coaching session to peers or colleagues. Open-ended feedback from respondents aids in interpreting the relatively lower levels of agreement linked to the session focused on vulnerability. Some respondents noted that the emphasis on vulnerability might have come too early in the overall sequence of trust-building. These remarks suggest there could be value in delaying the focus on vulnerability until teams have had more time to build trust by working on other key technical and relational strategies.

**Table 2:** Reactions to coaching sessions.

Session	*n*	I am satisfied with the coaching session just delivered	I would recommend this coaching session to peers or colleagues
		% Agree/Strongly agree	% Agree/Strongly agree
Input 1: Vulnerability	3	67%	33%
Input 2: Co-learning & demonstration of expertise	3	100%	100%
Input 3: Empathy-driven exchanges and responsiveness	3	100%	100%
Input 4: Bi-directional communication and frequent interactions	2	100%	100%
Input 5: Quick wins	3	100%	100%

*n* represents the number of valid responses provided by participants.

Data from qualitative interviews also yielded themes related to participant reactions to the training and coaching sessions, offering additional insights about the feasibility and acceptability of training content. For one, participants described how training sessions covered topics that were “*familiar*” (e.g., communication, vulnerability) but enhanced the team's capacity to act on these topics through specific tools and activities. Participants also shared that having outside facilitation for these meetings was helpful and that the work that took place in these meetings to build team cohesion and trust did not “*feel like work*.”

In terms of constructive feedback, building on a point raised in open-ended survey responses, some participants further noted in their interviews that vulnerability as a relational strategy for trust-building was highlighted too early in the overall sequence of the training and coaching sessions. Without having a more solid relational foundation as a team and with the trainers, the activities related to vulnerability felt premature. As a result, some participants suggested that content related to vulnerability be placed later in the overall training and coaching sequence. Team members also discussed feeling uncertain about the training content and the “*journey ahead*” for building trust. Several team members shared that their uncertainty contributed to feeling “*uncomfortable for the first couple of sessions.”* However, all participants shared that as their understanding about the purpose of the trust building sessions grew, the sessions felt more predictable and their comfort level and active participation in training sessions increased.

**Figure 3:**
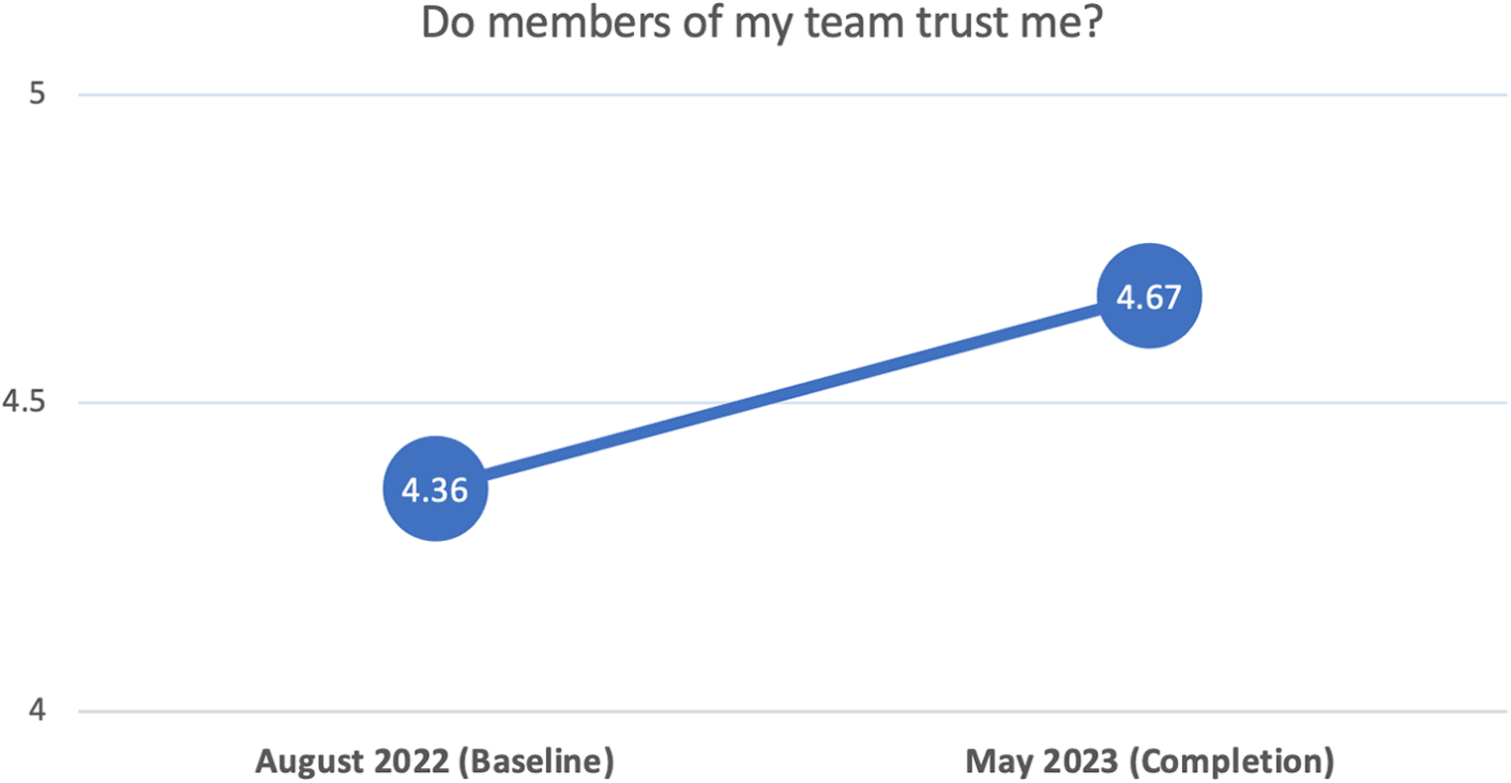
Visualization of change over time in perceptions of team trust in self. Outcome values range from 1 to 5.

In terms of RQ3, Figure 3 shows average scores estimated from 8 items that measured participants' perceptions that they were trusted by their team, with values ranging from a low of 1 to a high of 5. On average, participants reported significant increases over time in their perceptions that they were trusted by their team. Holding model covariates at sample-mean levels, the average rate-of-change from baseline (4.36) to completion (4.67) was 0.31 units (significant at *p* < .05); however, this value varied significantly across participants (i.e., significant random effect), such that one standard deviation (*SD*) below and above the average rate-of-change included the following range of values: −0.06 to 0.69. Thus, for some participants the rate-of-change was notably higher than average, whereas for others it was near 0. The figure simply captures the average rate-of-change in the sample.

**Figure 4:**
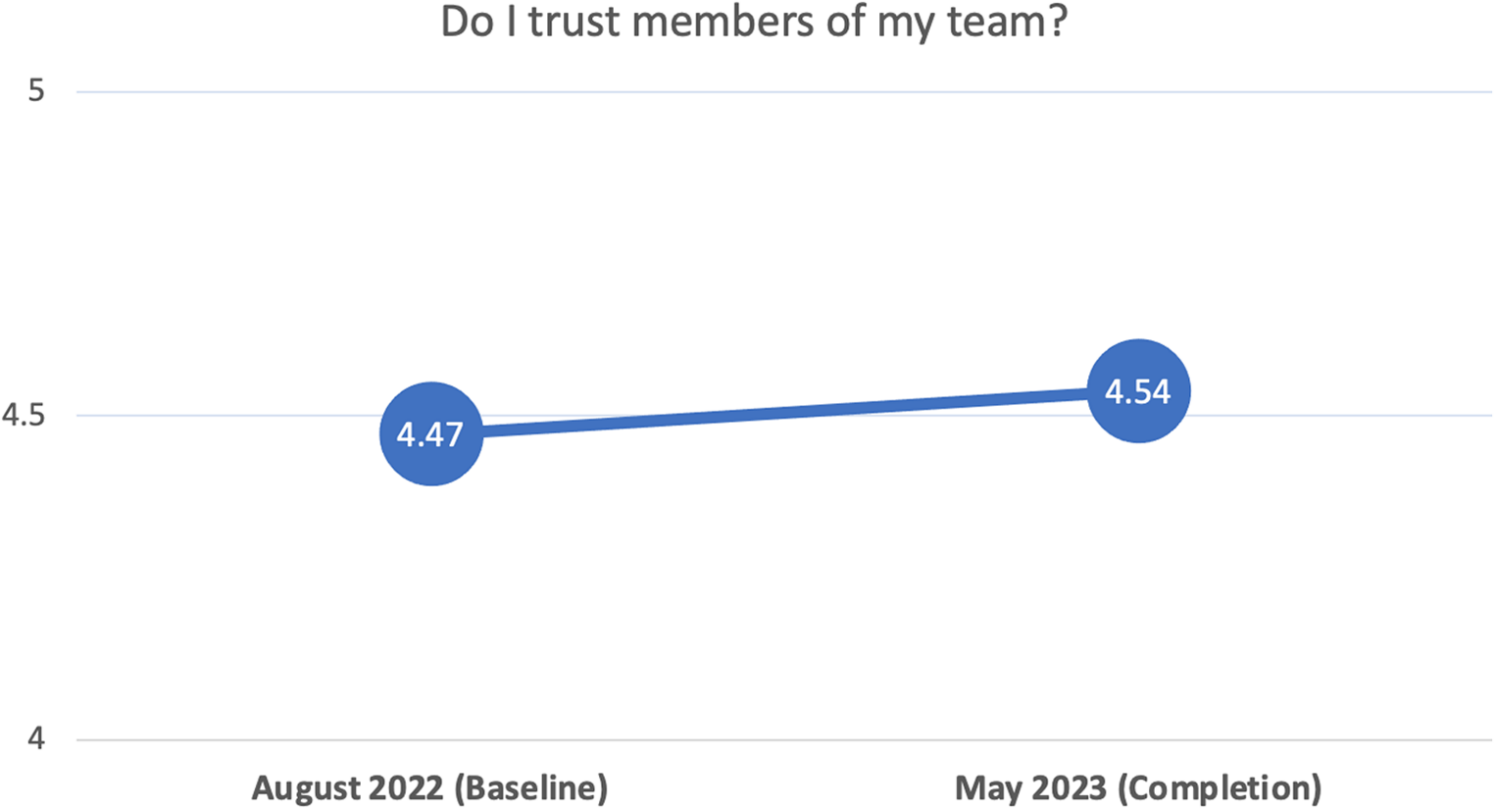
Visualization of change over time in perceptions of self trust in team. Outcome values range from 1 to 5.

Figure 4 shows average scores estimated from 8 items that measured participants' trust toward their team members, with values ranging from a low of 1 to a high of 5. On average, participants reported statistically negligible increases over time in the trust they had for their team. Holding model covariates at sample-mean levels, the average rate-of-change from baseline (4.47) to completion (4.54) was nearly 0.07 units (non-significant at *p* < .05); however, this value varied significantly across participants (i.e., significant random effect), such that one *SD* below and above the average rate-of-change included the following range of values: −0.29 to 0.42. The figure simply captures the average rate-of-change in the sample.

Turning to RQ4, findings from the qualitative interviews demonstrated support for the proposed theoretical model (see Figure 1). Participants described how the trainings on trust-building strategies triggered positive emotional responses among implementation team members which led to a sense that the focus on trust-building was a value-add to team members. Participants described how the training promoted a positive learning environment and contributed to increased trust among team members. Trust among team members subsequently contributed to team members reporting increased motivation to support implementation efforts, and a greater sense of capacity and opportunity to do so. Participants also reported feeling more resilient and committed to the work of the implementation team. Although longer-term outcomes related to evidence use were not feasible to measure at this early stage, some participants reported that greater trust contributed to more inclusive decision-making and data use among team members. Data-driven decision-making was described as central to implementation activities, with a focus on using evidence-based practices with fidelity. We now turn to granular findings related to specific elements in the proposed theoretical model.

### Positive affective response

Metz and colleagues describe positive affective responses to implementation support as positive emotional responses to interactions that happen within the context of implementation activities (18). These positive emotional responses may include feelings such as enthusiasm and joy and are hypothesized to contribute to positive relationships among implementation partners and team members. Interview findings supported the assumption that the use of relational and technical strategies would contribute to positive affective responses among team members. Team members described the trust-building trainings as “happy,” “freeing,” “enjoyable,” “calming,” “engaging,” “centered,” “peaceful,” and “dynamic.” Implementation team members described a sense of apprehension at first, not knowing what to expect as part of the trust-building work but emphasized the “learning environment” created by the trainers, which increased their comfort and excitement engaging in trust-building activities. A team member shared “*I went from being what is this to oh, today is [training] day. I wonder what we're going to talk about and what today's session's going to be like. And then it became something to look forward to.”* Additional team members emphasized that the willingness of trainers to be “*vulnerable and engaged helped to build trust with the team members.”*

### Perceived value add

The value-add for participating in the training refers to the extent to which end users of the training perceived benefit in training content. Perceptions of value are commonly explored in participatory research studies where researchers assess the extent to which engaged partners believed these activities produced added value economically, strategically, or relationally (46). All team members perceived the trust-building training and coaching as a value-add to the work of the team. Specifically, team members reported deepened relationships and increased empathy among each other, the usefulness of the material to use in other team meetings and their work more generally, an increased self-awareness of how they could contribute to a positive implementation environment, and an appreciation for the focus on the “non-technical” aspects of implementation and evidence use that can make or break implementation success.

#### Deepening relationships and empathy

Participants reported that a value-add of the training was increased empathy for the different perspectives of team members who played a different role than they did in the work (e.g., public agency staff reporting increased empathy for private service providers and vice versa). Private service providers often compete with each other for contracts with the public agency. In this case, private service providers reported feeling more empathy with each other for the first time. One team member shared “*I felt like it helped us to grow and for me to be more empathic to the other providers in the training.”*

Participants shared that the trainings allowed them to “*explore deeper relationships and teaming*” together and “*brought the implementation team together in a way that felt like a bonus*.” Participants appreciated setting aside explicit time for relationship-building. “*[Training on trust] embedded into your regular teamwork. It was this kind of special time that was allocated and that one thing we've also benefited from is having outside facilitators.”*

#### Usefulness of training material for future teams

Many team members reported on the usefulness of training materials and their use of these materials with other teams. Participants reported that “*tools were useful;”* they planned to “*apply this content on other teams, with other initiatives, and with other implementation efforts;”* and training materials would be “*value-add in the future.”* One participant shared that the trainings affected how she led her meetings now for all the work she oversees. Another participant described how the trainings “*influenced onboarding processes for the project, with a focus on trust-building and using exercises from the team meetings.”*

#### Self-awareness on roles and contribution to a positive implementation environment

Participants described how the trainings on trust-building made them more aware of themselves in relation to others on the implementation team, noting that the trainings increased how cognizant they were of other people's emotional experiences. A participant described their awareness of how this training could influence specific roles in support of implementation noting, “*This work may be the tip of the iceberg. [We can] delve into that some more for team cohesion and cultivating trust among the peer navigators, as well as the providers. You know those [supervisors] that are supporting peer navigators, there's an avenue and an opportunity there.”*

Another team member shared how this training influenced how they thought about their own role stating, “*[Implementation] support should be proactive and we [should] anticipate rather than be reactive to implementation challenges.”* Another team member also reflecting on their role shared “*Taking a step back to identify whatever my team was going through at the moment. Even though I had fifty things over my desk, that doesn't mean that I have the most things going on. And so, I think it just helped me keep being extra patient even though that is really hard sometimes.”*

#### An appreciation for the focus on the “non-technical” aspects of implementation

Team members commented on how the trainings on trust-building offered something more than “*just dealing with the technical aspects of implementation.”* A participant offered “*[Trainers] were shining faces apart from what we were having to deal with in the nitty gritty of the budgets and the staff. It was almost like a refresh.”* Team members described how the trainings resonated for them, as they understood that relational processes would influence whether implementation outcomes were achieved and commented that trust-building should inform all onboarding processes for the project, “*with a focus on trusting-building and using exercises from the team trainings.”* As implementation team members looked ahead to supporting peer mentors in their role, they described how the trainings provided scaffolding for supervisors who will need to “*demonstrate vulnerability with peer mentors”* as a way to facilitate psychological safety for peer mentors, who are the crux of the evidence-based intervention for youth in foster care.

### Psychologically safe learning environment

A psychologically safe learning environment refers to a space where people feel safe to take interpersonal risks, speak up and share concerns, and lift up new ideas without fear of reprisal (47). Without a culture of safety, a culture of silence can keep team members from identifying and addressing challenges while creating an illusion of implementation success. The theoretical model posited that trust-building strategies would contribute to the development of a psychologically safe learning environment. Interview findings supported this assumption, with team members emphasizing that setting aside time for trust development created a safe learning environment. One team member shared “*I do believe that the discussions we had within the [training] sessions really helped me to build strong relationships with this team, and comfortable relationships. Ones where I am, hopefully, creating an environment where people can feel comfortable being vulnerable and engaging with me in a way where we can [all] do that.”* Participants highlighted that training and coaching sessions gave team members time to learn together, the ability to brainstorm ideas, and the chance to discuss challenges.

Participants described how their sense of safety would contribute to the safety of peer navigators delivering the intervention, noting that if they (public and private agency staff) felt comfortable speaking up in team meetings, they would serve as a model for peer navigators to also feel safe to contribute ideas, express concerns, and ask questions. One team member shared “*After these [training] meetings, when we had to be present in some of the tougher implementation team meetings, I did feel like there was a sense that we could speak up or disagree.”* Another team member who represented a private provider agency commented “*If we don't understand things, [team leaders] are willing to meet with anybody on the team as much as necessary. They go over the information. I think all the relationships that we have are very positive and supportive. If I don't understand something, I feel comfortable enough to ask, ‘Hey wait, you know, what does that mean? Can you explain that to me?’ And there's not judgment. Everybody's just very willing to work together.”* Both public and private agency leaders and staff described how trusting relationships helped to level the playing field where they felt “we can all do this together. We are not just a provider or just a nonprofit.”

### Trusting relationships

Interpersonal trust is defined by McAllister as follows: “the extent to which a person is confident in and willing to act on the basis of the words, actions, and decisions of another” (48). Trusting relationships are further described as centered in vulnerability where the beliefs or expectations of individuals in the relationship are that actions will cause no harm and will provide benefit (39, 49–51). The theoretical model hypothesized that if the trust-building sessions (a) evoked positive emotions among team members, (b) resulted in perceived value-add for the team, and (c) created a safe learning environment, there would be an increase in trust among team members. Participants agreed with this assumption, emphasizing that trust among team members grew because the training sessions provided an opportunity to explore relationships and different perspectives, have reflective conversations, connect in new ways, and accelerate empathy for different roles in the face of implementation challenges.

Team members described how training sessions offered—for the first time—a chance for provider agencies and the public state agency to connect and have honest conversations. “*I don't want to speak for any of the providers, but I know one of the providers I had met with in the breakout sessions had said that it felt like they were able to connect with the state [agency] staff and really give open and honest feedback.”*

Team members described how trusting relationships promoted an “*ease of implementation,”* remarking *“I think that you know as you're starting to rely on one another, it builds that environment…you are more trusting in your peers, you're a little bit more willing to ask for help or just talk in general versus an environment like in the beginning when we didn't really know each other, when the program was new, and we were all trying to figure out what we do to make implementation of this program happen.”* Another team member reflected *“You are more trusting in who you're working with, and then it's easier to implement what you need to.”*

Team members highlighted a shift that took place from the common implementation challenge of team members not speaking up, to a more positive implementation climate where team members felt a sense of comfort sharing their perspectives. “*I think it helps you to see when people are feeling vulnerable about what they're going to say in the group. There's power and control in a lot of what is said. So…people are trying to control what they're saying or not saying. But then when we discussed empathy, there's a guard that gets let down. And people are feeling like they can be themselves more because they're being empathic.”*

Team members also commented that trusting relationships sustained the team's work in the face of an implementation challenge. One team member reflected “*I definitely feel like being able to implement a program with people and work so closely with them builds that community where trust is also a part of it…being able to follow through with things, being able to give [team members] the space to say what their needs are and actually for us to follow through with that, it is a huge trust builder.”* Another team member commented, *“You are more trusting in who you're working with, and then it's easier to implement what you need to get done throughout the day.”*

Trusting relationships allowed team members to be more discerning in their approach to addressing implementation barriers. A team member commented, “*I don't think we have enough opportunities with the implementation team to authentically work on a lot of these things.”*

Another team member reflected on relationships noting “*[team relationships] help me stop and think about how to approach this person or this situation. What are the things I need to take into consideration instead of being reactive to everything that's coming?”*

### Capability, opportunity, and motivation

Capability, opportunity, and motivation represent the behavior change framework described by Michie and colleagues (28). Capability includes both knowledge and skills to do something new, whereas opportunity includes both available resources and social conditions amenable to trying out something new. Finally, motivation refers to both the belief that doing something new or different will have a positive impact, as well as the established routines and habits that encourage new ways of work. The theoretical model posited that trusting relationships contribute to changes in capability, opportunity, and motivation, eventually leading to improved implementation and evidence use. Qualitative findings supported this assumption, with all interview respondents making connections between their team relationships and sense of safety on the team to increases in their sense of capability, opportunity, and motivation to support the implementation of the evidence-based peer mentoring program.

#### Capability

Team members described how learning from each other and understanding the range of perspectives on the team increased their sense of capability in contributing to the work of the team. One team member noted “*…We can have this time to learn something together, how it can be applied, and then relate it back to the work. So, that in itself was really great. So those of us who were able to participate [in training sessions] had a better understanding of teaming and the challenges that may arise from it, so we weren't surprised when there were challenges.”* Another team member described how the training sessions “*created an atmosphere of cohesiveness”* among team members so that team members could more effectively support implementation efforts. The team member shared “*I definitely felt more [capable]. I personally felt more valued in my role and what I was supposed to do, so it made sure I did what I needed to do, and there was definitely..a lot of support and encouragement, and I think everyone was just very excited for this new program to get started so everyone really supported each other.”*

Another team member described how each of the training topics contributed to an increasing sense of capability*. “From empathy, co-learning, bi-directional communication, frequent interactions, feedback loops, and maintaining openness. I think all of those [training topics] work to help increasing capability. I think when there's open dialogue..and intent to bring attention to that, it helps support positive relationship-building, and it helps in moving that work forward…. There's more clarity, people are more comfortable. I see a huge benefit to it.”*

#### Opportunity

Team members reported that the trust-building sessions created an opportunity to give voice to private provider agencies who often don't often have a voice with the state public agency. One team shared “*Input isn't often asked for, or considered, so this [was an] opportunity to build these relationships…come together as a team, as a provider to be able to give input on the strengths or challenges that we should consider in the implementation of this program, or even in the development and creation of this program.”*

Another team member emphasized “*It gave us as providers an opportunity that we don't always have… to have a voice, you know? And then through the [training] sessions it gave us an opportunity to build relationships with different parts of the team. The fact that we all came together, including the program leads from [public agency] from the different offices, and the researchers, and the model developers, so we really could see from start to finish how this is coming about..was really helpful. It gives context to everything that's going on, and we don't always have context as to why certain things are happening, or why certain decisions are being made. So then sometimes things get lost in the implementation of programs.”*

Training sessions provided team members from private provider agencies with a unique opportunity to gain perspectives on how and why the state agency operates as it does. *“[The training sessions] gave really good opportunities to talk about how the state operates as fast as the state's going to operate and sometimes that puts a lot of stress on the providers, and I was really able to gain their perspective in that sense.”*

#### Motivation

Team members emphasized how trusting relationship also motivated them to support implementation. A team member shared “*It motivated [all team members], they learned about what other agencies were doing and they were able to give feedback to what they were doing to individuals who genuinely wanted to know and genuinely cared about this program because they're all invested, involved.”* Setting aside the time to build relationships on the team was mentioned by many team members as an opportunity to understand and participate in decision-making.

One team member shared “*Having these opportunities gave us a full understanding of what was someone's thinking behind this part of the implementation process, and then we can make a connection to that. Truly understand why we are doing this work. I think it also helps motivate us to continue to do it.”*

### Commitment and resilience

Metz and colleagues described commitment and resilience as bringing patience and willingness to challenge the status quo during the implementation process; creating readiness for change; and investing in building effective teams (11). The theoretical model emphasized that as team members demonstrate increased capability, opportunity, and motivation to actively support implementation efforts, they will also feel more committed and resilient in the face of implementation barriers, challenges, or setbacks. Team members demonstrated support for this theoretical assumption, reporting increased buy-in to team activities and continuing to meet as a team in the future as well as increased sense of capability and commitment to overcome implementation challenges and an increased resilience to address challenges noting *“I think as a supervisor it helped in-house with my own team because when we did hit those little bumps, I felt like I was able to identify a bit more with the emotion that was going on with the bump.”*

Another team member described “*We want the peer-to-peer mentoring program to succeed, right? So, when you have the opportunity to focus on how to improve, and how to better work together to have more cohesion, to have more openness, to have more dialogue and communication, I think it helps us to certainly find a space to become more energized, and more committed to what we want to accomplish.”*

Team members emphasized that relationships and connections support commitment and resilience in the work. One team member shared “*In Brené Brown's book ‘Braving the Wilderness’ she talks about the health impacts of not being connected..the role of relationships is absolutely critical in everyone's life. I appreciate the opportunity to be a part of these [training] sessions just because we can forget that often, especially when we have stressful days because that's when we're pushing people away. So just having these reminders, having opportunities to build relationships with different people, and learn to strengthen the ones that we have. This is why we do the work that we do. So, I've appreciated this.”*

### Use of evidence and implementation outcomes

As articulated by the William T. Grant Foundation, use of evidence refers to the multiple ways that research can be used to help clarify a problem, influence decision-making, improve outcomes, or to build trust or educate partners. The theoretical model hypothesized that as team members became more committed to implementation efforts, they would be better equipped to achieve implementation outcomes and support ongoing use of evidence. In this case, team members demonstrated commitment to supporting implementation of the evidence-based peer mentoring program to improve outcomes for youth in foster care. Team members also demonstrated a firm commitment to data use, inclusive decision-making, and the use of small tests of change for ongoing improvement.

Data were described as the starting point for all decision-making (data as the convener of the implementation team meeting) with a growing openness on the part of team members to ensure that data were interpreted from multiple perspectives before making decisions based on early implementation findings (e.g., fidelity score). A team member described this process, “*How does everybody feel? Do they have any ideas? Do they want to change it? Is this okay? You know, and then us as the implementation team meet and discuss [the data], so everyone's opinion was really taken into consideration.”* Another team member described how decisions were not made quickly by the team and demonstrated an evolving commitment to do “homework” and come back together. The team member noted how different this was from previous experiences where team members may say “*Okay, we need to make a decision in 10 min about something that's hugely impactful for the practice of the program.”* Slowing down decision-making by reviewing data demonstrated an emerging commitment to evidence use by team members.

## Discussion

Core aims of the current study included assessing the feasibility (RQ1) and acceptability (RQ2) of a training and coaching approach that builds the skills of leadership and staff in a public child welfare system who support implementation of evidence-informed initiatives to foster trusting relationships among each other and with community partners. Our findings suggest that the training and coaching approach was feasible and acceptable, as evidenced by attendance rates and participants' reactions to training and coaching sessions. The generally good attendance at training modules highlights the value in embedding training modules within existing team meeting structures. Coaching sessions with team leaders were intended to help mitigate challenges associated with turnover rates, in that team leaders would have the skills to continue to use the trust building activities with team members. While team leaders shared their commitment to using the trust building activities with additional teams, future research is needed to better understand how to embed trust building into day-to-day activities for public agencies. Additional studies are also needed on how to scale trust building into implementation efforts across public systems with little resources to allocate time or money to these types of activities. Train the trainer models are a potential method for low-cost replication.

Our findings also indicate the potential value in placing content focused on relatively high-demand relational trust-building strategies, such as vulnerability, later on in the overall sequence of content delivery—although there is likely notable variability in preference among participants on this front. Future research could explore further the optimal sequence of content delivery. In any case, our findings emphasize that in addition to having strong, theory-driven content, trainers need to actively and authentically model the trust-building strategies being taught to optimize the efficacy of the training and coaching approach.

Another core aim of the current study was to assess whether the training and coaching approach contributed to the development of trusting relationships among members of implementation teams who support implementation of evidence-informed initiatives in a public child welfare system (RQ3). On this front, our findings yielded some initial support. Particularly with respect to one's perceptions that members of their team trust them, our findings showcased significant average gains from baseline to the completion of the training sequence. Gains were not as pronounced in terms of one's report of their own trust toward members of the team. It is important to note that levels of trust on both of these fronts began quite high at baseline, potentially producing burdensome ceiling effects, such that many participants might not have had much room for gains over time. Another dynamic of our study context that could have obscured efforts to accurately measure trusting relationships was the malleability of team membership over the duration of the project. That is, team membership expanded and contracted over time as a result of the needs and tasks of the implementation team and their plans to expand implementation efforts. As a result, some participants might have experienced difficulty in evaluating the trust dynamics of the team from one time-point to another.

These issues signal promising opportunities for future research, whereby our training and coaching approach could be evaluated in contexts with relatively stable teams and/or contexts marked by low baseline levels of trust, enabling assessment of whether this starting point moderates the efficacy of the training and coaching approach in promoting trust-building over time. Such evaluations could be bolstered further through the application of experimental designs [e.g., randomized control trials (RCT)]. Indeed, our intentions for the current study were not to draw firm causal inferences, as we cannot rule out that any gains in trusting relationships would have occurred at the same rate naturally without engagement in the training and coaching approach. RCTs could produce a more solid counterfactual framework for assessing the potential causal impacts of the training and coaching approach on trusting relationships and other outcomes posited in the proposed theoretical model. Of course, RCTs of this sort would require notable resources. Future research could also attend to plausible moderators of training efficacy, deepening our understanding of for whom and under what conditions (e.g., virtual format vs. in-person format) the training and coaching approach is most or least efficacious.

This study also highlights important opportunities with respect to measurement. Although we adapted an existing measure of trusting relationships (which performed well in our sample), we do see value in future efforts to develop and refine measures of trusting relationships that might be especially well suited for implementation contexts. Metz and colleagues have offered additional points about measures related to the theoretical model informing the current study (18). There also could be value in assessing constructs like trust and trusting relationships using a relatively more intensive data collection schedule. For example, intensive longitudinal approaches could be used to assess focal constructs on weekly or even daily intervals, enabling the assessment of perturbances in construct levels on a relatively moment-to-moment basis (52).

A final core aim of the current study was to assess whether the development of trusting relationships among members of implementation teams who support implementation of evidence-informed initiatives in a public child welfare system actually improves the use of evidence for decision-making and the implementation of evidence-informed initiatives (RQ4). Findings from qualitative interviews yielded rich themes that supported many of the elements highlighted in the proposed theoretical model, which intends to foreground the specific mechanisms by which trusting relationships promote evidence use and implementation. To be sure, we view these findings as nascent and preliminary. We look forward to ongoing and increasingly robust efforts to empirically corroborate and refine the proposed theoretical model.

Ongoing research on the strategies that promote trust among team members is an important contribution to implementation science. Indeed, many funders and calls for proposals encourage the use of multi-disciplinary implementation teams and have expectations related to high-functioning implementation teams that include diverse perspectives in planning and problem-solving activities (35). This current study speaks to the role of trust on implementation teams and provides practical strategies—both relational and technical—for trust-building that are replicable and can be further tested in different service contexts.

The development and testing of the training and coaching sessions were driven by theory that posits relationships emerge from positive affective experiences which foster trust, resilience, and commitment in the face of implementation challenges (relational cohesion theory) (22, 23) and that increased empathy contributes to a sense of mutual interdependence reinforcing positive affective responses among team members (relational cultural theory) (26). This study offers emerging insights into the role of emotional experiences of team members in developing trusting relationships and increasing their motivation and perceived capability to effectively contribute to implementation efforts.

Emotional responses to implementation efforts are often overlooked in the implementation science literature, including the identification of strategies that can promote positive emotional experiences among team members and implementation partners. An exception to this is the Theoretical Domains Framework (TDF), which includes the role of emotions in promoting the behavior change needed for implementation efforts to succeed. The TDF defines emotions as “a complex reaction pattern, involving experiential, behavioral, and physiological elements, by which the individual attempts to deal with a personally significant matter or event” (53). This study underscores the importance of affective responses to implementation activities conducted within teams and the role that positive emotions can play in increasing the commitment of team members to address implementation challenges. This study also points to specific trust-building strategies that can be used to activate important positive affective responses to change, increasing the likelihood that teams will be successful.

### Study limitations

The current study seeks to provide replicable strategies for trust-building among implementation partners. However, this study is limited by its exploratory nature, the small sample size, and the single and particular setting (public child welfare setting). Future replication studies can address whether trust-building strategies are generalizable to a range of service settings, and longitudinal studies can better assess how trust-building contributes to long-term implementation outcomes and population impact.

As noted earlier, the theoretical model limits the extent to which the current study can focus on addressing power structures in implementation activities. Future studies can test whether specific facilitation techniques that make power structures more visible and protect all voices can have further benefit, and whether identifying the influence that different partners have more explicitly can help to address power differentials in team processes. In addition, longitudinal and case study designs would allow for more in-depth assessments of how, and through what mechanisms, trust permeates various levels of an organization including case planners, supervisors, mangers, and senior leaders.

The current study was guided by a previously published theoretical model for building trusting relationships to support implementation and evidence use (18). The purpose of this study was to test the assumptions of this theoretical model in service settings implementing a change effort that involved wide scale use of an evidence-informed program or practice. However, it can be argued that alternative conceptual models related to the development of relationships on implementation teams and the outcomes associated with relationally based teamwork could also be considered to support interpretation of the findings. Specifically, implementation teams have been documented as a powerful resource for supporting implementation efforts (54, 55). High performance implementation teams have been described as operating with a common purpose to continuously improve and reach goals. Team members work together embracing a sense of mutual accountability for the work (54).

Cooperative characteristics of high performing implementation teams typically don't emerge on their own but involves a set of strategies to achieve interdependence of team members. These strategies are often relationally based and include building trust, managing healthy conflict, achieving commitment, embracing accountability, and focusing on results (56). Recent research on teams found that team interdependence (i.e., sharing ideas and resources and focusing on joint outcomes) was related to positive implementation climate, increased reach with focus populations, and reduced time to implementation. This research also pointed to the importance of relationships on teams finding that positive affective team functioning (i.e., perceptions of trust, relationships and respect among team members) was related to specific implementation outcomes including achieving acceptability and buy-in for the intervention, supporting a strong contextual fit and appropriateness between interventions and service settings, and ensuring that implementation strategies were feasible to use to promote implementation progress (57).

Further, emerging research in implementation science on the role of relationally based strategies speaks to the importance of understanding the theoretical and practical role relationships play in implementation efforts (12, 58). Indeed, Metz and colleagues foregrounded an important question in implementation science related to whether high quality relationships among implementation partners serve as a moderator for all implementation strategies used to promote implementation progress and the achievement of implementation and population outcomes (12). In any case, there will be value in selecting or adapting measures that, with validity and reliability, measure distinct relational constructs of interest. Some specific measures on this front are highlighted by Metz and colleagues (18). This current study was funded to test a specific theoretical model for trust-building and evidence use. However, future research on trust-building can seek to disentangle the mechanisms of change related to whether and how trusting relationships and/or the interdependence of high functioning teams contributes to implementation outcomes.

## Data Availability

The raw data supporting the conclusions of this article will be made available by the authors, without undue reservation.
