# Supplementary material for: Building trusting relationships in teams to support evidence use and implementation in human services: feasibility and acceptability of a training and coaching approach
Source: Front Health Serv. 2024 Dec 10;4:1353741. doi: 10.3389/frhs.2024.1353741 (PMC11670258; doi:10.3389/frhs.2024.1353741)
Supplement: Supplementary file 1 [file Datasheet1.docx]

**Summary of Trust-Building Strategies and Exercises**

| Session | Strategy | Activity |
| --- | --- | --- |
| Oct. 2022 | Authenticity (Relational) | **One-to-One activity**: The purpose of this activity is to understand the motivations and underlying values of teammates, specifically as it related to what led them to work in child welfare. Team members were moved into breakout rooms in groups of three (with diversity of perspectives by agency/role in each group). Each member of the small group rotated roles between interviewer, interviewee, and observer. Interviewers asked what led them to work in child welfare, interviewees shared their motivations, and observers identified values they heard in the exchange. |
| Dec. 2022 | Vulnerability (Relational) | **Full group discussion**: Team members reflected on the following questions:   - If part or all of your job is minimizing systemic vulnerability, how does this shape your thoughts and feelings about the importance of relational vulnerability? - On my team, the messages and expectations about the concept of vulnerability include:   **“What you need to know about me to really know me” exercise**: The purpose of this exercise is to demonstrate vulnerability with a team member(s). Team members were moved to breakout rooms in pairs and completed this phrase, sharing something about themselves (typically professional). The other team member repeated, I hear you,” after each share/statement. The team members then switched roles. |
| Jan. 2023 | Co-learning (Relational)  Demonstration of expertise (Technical) | **Co-learning exercise**: The purpose of this exercise is to allow team members to engage in co-learning and also demonstrate the expertise and experience they contribute to the implement effort. Team members were moved into breakout rooms in groups of three. In their small groups, team members shared responses to the following questions:   - What do you think is going well? - What really worries you? - What would it look like for you, in your role, to effectively support implementation? - What support do you need in this role? |
| Feb. 2023 | Empathy-driven exchanges (Relational)  Responsiveness (Technical) | **Empathy mapping exercise**: The purpose of this exercise is to consider what the emotions, experiences and perspectives of other implementation partners or stakeholders may be. As a full group, team members considered what youth mentors may be saying, thinking, doing and feeling as it related to implementation of the peer mentoring model. Team members reflected on how empathy maps can help to gain perspective on teammates and increase trust. |
| Mar. 2023 | Bi-directional communication (Relational)  Frequent interactions (Technical) | **Small group reflection**: The purpose of this activity is to reflect on the impact of strong (or weak) communication on implementation efforts. Team members were moved into breakout rooms in random groups of three. They were asked to remember a time when they were part of an implementation effort and communication among team members or between teams was bi-directional and frequent, and to share responses to the following questions:   - What did that look like? - How did that communication contribute (or not) to your attitude toward implementation? |
| Apr. 2023 | Quick wins (Technical) | **Quick Wins Jamboard activity**: The purpose of this activity is for team members to reflect on early signs of progress for implementing the peer mentoring model. Team members were asked to share reflections:   - What are early signs of progress you’d like to look for as a team? - What early signs of progress have you observed so far? |
