# Supplementary material for: Building trusting relationships in teams to support evidence use and implementation in human services: feasibility and acceptability of a training and coaching approach
Source: Front Health Serv. 2024 Dec 10;4:1353741. doi: 10.3389/frhs.2024.1353741 (PMC11670258; doi:10.3389/frhs.2024.1353741)
Supplement: Supplementary file 2 [file Datasheet2.docx]

**Interview Guide Questions**

| 1. Consider how you felt about the **training sessions**. What emotion words best describe your experience(s)? 2. Overall, how did the **training sessions** influence your team’s ability to build relationships with each other?    1. [Possible probe] What training session(s) made the biggest difference?    2. [Possible probe] Did you sense your *trust of others* on the team growing over time? How so?    3. [Possible probe] Did you sense others’ *trust of you* growing over time? How so? 3. In what ways, if any, did the **training sessions** affect your empathy for team members or other stakeholders involved in the implementation effort? 4. In what ways did the **training sessions** influence the team’s effort to implement the evidence-based peer mentoring program? 5. In what ways did **relationships among team members** help the team support implementation of the peer mentoring program? (Capability)    1. [Possible probe] How would you describe the team’s shared goals? *[knowledge]*    2. [Possible probe] Do you believe the team has the capacity to support implementation of the peer mentoring program? *[capacity]*    3. [Possible probe] How confident are you that the team can support implementation of the peer mentoring program? *[confidence]* 6. In what ways did **relationships among team members** create an environment that enabled the team to support implementation of the peer mentoring program? (Opportunity)    1. [Possible probe] How are research findings and data discussed among team members? *[collaboration]*    2. [Possible probe] Do team members engage in discussion regarding how research findings and data might be applied to decision-making? *[safety culture]*    3. [Possible probe] Do team members speak up with ideas, questions, and concerns about research findings and data presented to the team? *[safety culture]* 7. In what ways did **relationships among team members** energize the team to support implementation of the peer mentoring program? (Motivation) 8. [Possible probe] How do team members use research findings and data to support their ongoing learning? *[learning]* 9. [For team leaders] What impacts did the **coaching sessions** have on anything we have discussed so far? 10. Can you share an example of a decision that the implementation team made and describe the process by which the decision was made? 11. What else would you like to share that we haven’t had a chance to discuss yet? |
| --- |
